# Potential Benefits of Tryptophan Metabolism to the Efficacy of Tocilizumab in COVID-19

**DOI:** 10.3389/fphar.2020.00959

**Published:** 2020-06-19

**Authors:** Maria Laura Belladonna, Ciriana Orabona

**Affiliations:** Department of Experimental Medicine, University of Perugia, Perugia, Italy

**Keywords:** COVID-19, tocilizumab, tryptophan catabolism, IDO1, cytokine storm syndrome

## Abstract

Tocilizumab has been proposed as a means of opposing hyperinflammatory responses in intensive care patients with COVID-19. Here, we briefly discuss the potentially multiple, synergistic mechanisms whereby tocilizumab might exert therapeutic activity, mostly focusing on the production of tryptophan-derived catabolites that would result from blockade of IL-6 signaling, as contextualized to the cytokine storm occurring in COVID-19 patients.

## COVID-19 Origin and Immunopathology

On March 2020, the viral disease COVID-19 sparked by severe acute respiratory syndrome–coronavirus 2 (SARS-CoV-2) began to spread to such an extent that the World Health Organization formally recognized the COVID-19 outbreak as a pandemic. SARS-CoV-2 is responsible for SARS that is less virulent, but far more contagious than those caused by SARS-CoV and middle east respiratory syndrome (MERS)-CoV (Prompetchara et al., 2020), two viruses also belonging in the Betacoronavirus genus. Several studies suggested that the main source of SARS-CoV-2 transmission to humans is represented by bats, natural hosts of a wide variety of CoVs. Such a transmission likely involved multiple intermediate hosts—including turtles, pangolin, and snakes—whereby the viral genome accumulated a series of mutations, ultimately permissive of the jump the human species (Giovanetti and Benvenuto, 2020). Spread through the respiratory tract by the spraying of droplets and *via* close contact, human-to-human virus transmission occurs through an interaction between the viral spike glycoprotein and the angiotensin-converting enzyme 2, which, abundantly expressed in lungs by type-2 alveolar epithelial cells, is exploited by SARS-CoV-2 as a receptor to enter the cell (Zhou et al., 2020). As reported by a recent Chinese epidemiologic study (Bi et al., 2020), while a majority of COVID-19 cases presents with mild (26%) or moderate (65%) flu-like symptoms, a few patients (9%), mostly the elderly and/or with preexisting disorders, will develop severe or critical conditions, rapidly culminating in an acute respiratory distress syndrome (RDS), septic shock, and coagulation dysfunction, all of which can be lethal (Huang et al., 2020).

Disease outcome seems to critically depend on timing and strength whereby innate immune responses against the virus are activated (Prompetchara et al., 2020). During an efficient antiviral response, sensing of viral RNA by innate immune cells—a first line defense against the pathogen—will induce the prompt expression of type I IFN, a key cytokine capable of suppressing replication and dissemination of the infecting pathogen at an early stage. In analogy with SARS- and MERS-CoV, SARS-CoV-2 viral proteins are thought to inhibit IFNα receptor signaling and thus escape the early response to type I IFN (Perlman and Dandekar, 2005). A subsequent, uncontrolled viral replication triggers the influx of neutrophils and monocytes/macrophages into the lower respiratory tract and hyperproduction of chemokines and proinflammatory cytokines in the local environment, including IL-6, IL-10, and TNFα, causing a “cytokine storm” (Huang et al., 2020).

Therefore, the more the virus is able to contrast type I IFN production (by delaying and weakening this response), the more an overwhelming inflammatory response will be mounted in the lungs, the main target organ of SARS-CoV-2 (Prompetchara et al., 2020). If a cytokine storm occurs, the ensuing cytokine release syndrome (CRS) is typically associated with severe, rather than moderate, COVID-19, with an immunopathology being characterized by high serum levels of cytokines, CD4^+^ and CD8^+^ T (but not B) cell lymphopenia, diffused alveolar damage, pulmonary hypertension, pneumonia, and acute RDS (Pedersen and Ho, 2020).

## Tocilizumab (TCZ) Therapy as a Means of Mitigating CRS in COVID-19

Among pro-inflammatory cytokines in a cytokine storm, IL-6 plays a key role in inducing acute inflammation, as the one observed in RDS. IL-6 is a monomeric cytokine produced by almost all stromal and immune cells in response to infection or tissue injury. In particular, neutrophils, monocytes, and macrophages release IL-6 and other proinflammatory cytokines (IL-1β and TNFα), as an event downstream of toll-like receptor sensing of pathogens (Zhang et al., 2020). IL-6 signaling occurs through two pathways: the cytokine binds the transmembrane type-I receptor IL-6R (i.e., the classical pathway, mediating regenerative and anti-bacterial protective effects), or it binds the soluble form of the receptor (i.e., the trans-signaling pathway, inducing inflammatory mediators). Both pathways converge on the association with the same transmembrane gp130-homodimer to assemble the active receptor complex, which exploits the JAK/STAT pathway for signal transduction (Rose-John, 2012).

The potent proinflammatory IL-6 cascade is regulated by natural inhibitors, interfering with the complete assembly of the functional receptor (gp130 soluble form), or with its signal transduction system (i.e., SOCS proteins). The recombinant human monoclonal antibody TCZ, directed against the IL-6R, mediates an inhibition strategy, preventing IL-6 binding to its receptor and therefore IL-6 biological activity. Preferentially interacting with soluble rather than transmembrane IL-6R, the biological drug mainly opposes inflammatory more than regenerative responses to IL-6 (Sheppard et al., 2017). Because of its peculiar action impairing IL-6–mediated pathogenic immunity, FDA currently approves TCZ for treatment of adult rheumatoid arthritis, large-vessel vasculitis, such as giant-cell arteritis, polyarticular juvenile idiopathic arthritis, systemic juvenile idiopathic arthritis, adult-onset Still’s disease, and the severe often life-threatening CRS induced by chimeric antigen receptor T cell therapy. Current clinical trials include vascular and muscular diseases characterized by strong inflammatory and autoimmune components, and specific autoimmune diseases, such as type 1 diabetes, as being evaluated in an extended clinical trial (EXTEND, NCT02293837). Awareness of IL-6’s potential role in COVID-19 immunopathogenesis prompted new clinical trials aimed at assessing TCZ efficacy in COVID-19.

## TCZ and Tryptophan (Trp) Catabolism

While awaiting a vaccine to constrain the pandemic, IL-6 blockade seems to be a rational therapeutic approach to contrast CRS associated with COVID-19. TCZ, a first-line drug in specific pathologies, proved to be effective and particularly safe for acute treatment as would required for blocking IL-6 in COVID-19. The efficacy of blocking IL-6 signaling in COVID-19 is currently under investigation, with still unexplored backstage. The use of TCZ in the treatment of COVID-19 pneumonia finds a sound rational basis in the inhibition of virus-triggered excessive and aberrant inflammatory responses, leading to fibrosis and lung damage. However, IL-6 neutralization might lead to a variety of immunomodulatory mechanisms potentially contributing to its therapeutic activity in COVID-19 treatment.

A powerful immunomodulatory pathway opposing hyperinflammatory responses is represented by Trp catabolism, which generates a plethora of immunoactive metabolites, collectively known as kynurenines (Grohmann et al., 2017). In immune cells, the conversion of Trp into l-kynurenine (Kyn; a non-proteogenic amino acid) is finely controlled by the enzyme indoleamine 2,3 dioxygenase 1 (IDO1). A tight correlation has been demonstrated between IL-6–driven inflammation and the defective activity of IDO1, compromising its role of immune homeostasis regulator (Orabona et al., 2012). IDO1 defect—and thus the impaired generation of immunoregulatory Kyn—is caused by the ubiquitin-proteasome system-mediated proteolysis of IDO1 protein induced by IL-6 (Orabona et al., 2008). Both murine dendritic cells (DCs) and human peripheral blood mononuclear cells (PBMCs), readily responding to inflammatory stimuli, can activate such a mechanism of IDO1 turnover for repressing its immunomodulatory activity and thus express a ready-to-start immunogenic phenotype. In hyperinflammatory conditions, aberrant secretion of IL-6, as a triggering mechanism of IDO1 proteolysis, leads to accelerated IDO1 proteolysis and to the knocking down of a potent immunoregulatory mechanism. In COVID-19-associated CRS, the immunoregulatory Trp-derived metabolites generated by IDO1 could be deficient and their absence could worsen the inflammatory events in COVID-19. IL-6-mediated IDO1 proteolysis has been previously reported as a pathogenic mechanism in autoimmune diabetes (Mondanelli et al., 2017), where it contributes to the onset of the autoimmune response against pancreatic autoantigens. TCZ was demonstrated to be beneficial in hyperglycemic NOD mice in an IDO1-dependent manner and to restore *in vitro* IDO1 activity in PBMCs from pediatric diabetic patients (Orabona et al., 2018), thus confirming the relevance of Trp metabolic pathway in the control of inflammation and the close interplay between IL-6 and IDO1’s activity.

In the control of hyperinflammatory responses, Kyn—the main byproduct of Trp metabolism—plays a major role as an endogenous agonist of the immunoregulatory Aryl hydrocarbon Receptor (AhR). As a result, a feed-forward loop constraining hyperinflammation is established, whereby IDO1 generates Kyn, an activating ligand for AhR that, in turn, will enhance IDO1 expression in DCs (Manni et al., 2020). Such a positive feedforward loop contributes to a state of endotoxin tolerance, protecting mice against further bacterial LPS challenge after a first exposure to LPS (Bessede et al., 2014). Notably, AhR is widely expressed in immune cells where the activated receptor can tip the balance regulatory and inflammatory T cells in favor of the former subset. Administration of AhR agonists alleviated immunopathology in various animal models, such as experimental autoimmune encephalomyelitis, experimental colitis (Quintana et al., 2008) and, recently, a model of pulmonary fibrosis (Takei et al., 2020). Kyn is also a potent vasodilator of systemic arteries and has been found to be significantly increased in pulmonary arterial hypertension patients. In chronic models of pulmonary arterial hypertension, Kyn exerted acute pulmonary vasodilatation in synergy with nitric oxide (Nagy et al., 2017).

**Figure 1:**
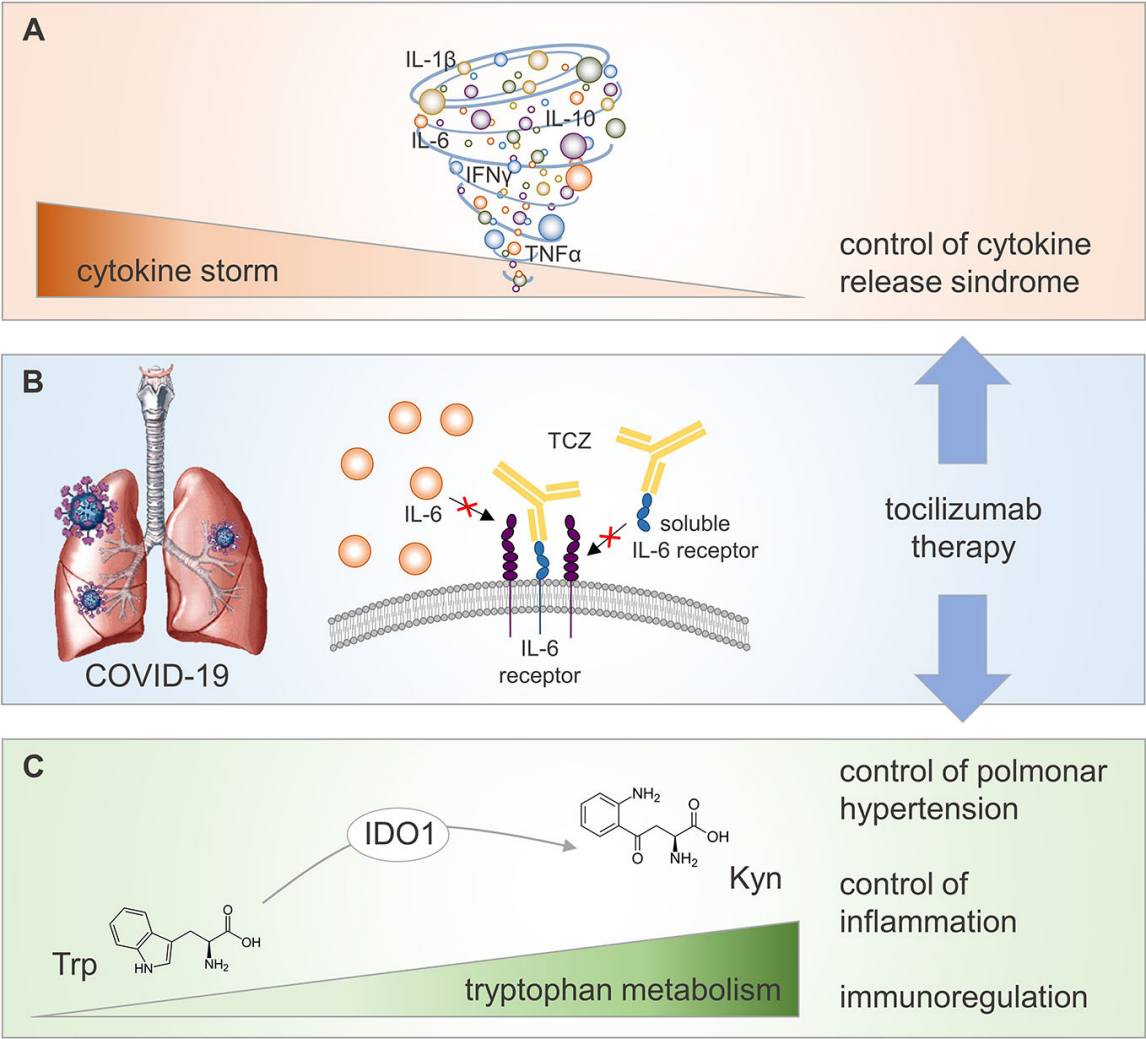
Potential regulatory mechanisms activated by TCZ therapy in COVID-19 patients. COVID-19 is associated to a CRS referred as “cytokine storm” **(A)**, whose reduction at lung level (the main target organ of SARS-CoV2 viral infection) may be achieved by TCZ therapy inhibiting IL-6 proinflammatory effect **(B)**. Neutralization of IL-6 biological activity by TCZ potentiates IDO1-mediated conversion of the amino acid Trp into its catabolite Kyn **(C)**. Increased level of circulating Kyn contributes to control pulmonary hypertension and inflammation, and to promote immunoregulation in COVID-19 patients.

Overall, the symptomatic approach using TCZ in COVID-19 pneumonia might involve still uncovered beneficial effects due to synergistic, yet distinct, mechanisms downstream of IL-6 signaling (**Figure 1**). Among those, the restoration of an active Trp catabolism, otherwise impaired by IL-6, might generate proper levels of Kyn for the control of pulmonary hyperinflammation and hypertension.

## Concluding Remarks

Several multi-center, randomized controlled trials for assessing the efficacy and tolerability of TCZ have been approved in patients with COVID-19 pneumonia. At the moment few data are available. A small clinical trial in China (ID: ChiCTR2000029765), recruiting 21 patients with severe or critical COVID-19, has shown good efficacy. The remaining approved trials are ongoing all over the world. Based on the infection emergence, strict eligible criteria were adopted in most studies for the recruitments of COVID-19 patients. However, few studies (i.e., COVIDOSE, NCT04331795) aim to titrate TCZ in non-critically hospitalized ill patients, in order to identify the optimal timing to treat them during the infection, to personalize TCZ therapy and hopefully avoid these COVID-19 patients intensive care. In addition to clinical responses (i.e., decreased mortality incidence, decreased fever, etc.) evaluated as primary outcomes, a series of biochemical parameters are also monitored as secondary outcomes, that is, levels of circulating IL-6 and C-reactive protein, leukocytes count, and oxygen saturation.

Solid published results demonstrated a close relationship between TCZ action and an increased Trp catabolism in different inflammatory settings driven by IL-6 (Orabona et al., 2008; Mondanelli et al., 2017; Orabona et al., 2018), suggesting the possibility and relevance of monitoring Trp metabolism in COVID-19 patients on TCZ therapy. The ratio between circulating Kyn and Trp (Kyn/Trp) in patients’ sera might represent a biomarker for assessing IDO1 activity and a surrogate biomarker for tracking immunoregulation mediated by Trp catabolism.

In conclusion, IL-6–mediated inflammation in COVID-19 might affect Trp catabolism, as observed in autoimmune diabetes and other experimental conditions. TCZ treatment might restore a proper IDO1 activity, providing immunoactive Kyn as a ligand for AhR-dependent immune regulation, including the fostering of T-regulatory cell responses. This might alleviate local and systemic hyperinflammatory responses in COVID-19 patients. Currently, TCZ is clinically trialed for targeting IL-6R in COVID-19 patients. These studies offer the opportunity of molecularly dissecting synergic mechanisms capable to mitigate COVID-19-related symptoms, opening the way for the setting of innovative combined therapies.

## Author Contributions

MB wrote the manuscript and edited the figure. CO wrote and revised the manuscript.

## Funding

This work was supported by the Italian Ministry of Education, Universities and Research (PRIN2017- 2017BA9LM5 to CO).

## Conflict of Interest

The authors declare that the research was conducted in the absence of any commercial or financial relationships that could be construed as a potential conflict of interest.
